# Clinical characteristics and management of acute generalized exanthematous pustulosis with haemodynamic instability

**DOI:** 10.1002/ski2.74

**Published:** 2021-11-15

**Authors:** M. O’Brian, C. L. Carr, C. Thomas, A. R. Dominguez, M. Mauskar

**Affiliations:** ^1^ University of Texas Southwestern Medical Center University of Texas Southwestern Medical School Dallas Texas USA; ^2^ Department of Dermatology University of Texas Southwestern Medical Center Dallas Texas USA; ^3^ Department of Internal Medicine University of Texas Southwestern Medical Center Dallas Texas USA; ^4^ Department of Obstetrics and Gynecology University of Texas Southwestern Medical Center Dallas Texas USA

## Abstract

**Background:**

Acute generalized exanthematous pustulosis (AGEP) is a severe pustular drug eruption with rare reports of haemodynamic instability.

**Objective:**

To describe the clinical characteristics, management, and outcomes of patients with AGEP‐associated haemodynamic instability.

**Methods:**

This retrospective case series identified adult patients diagnosed with AGEP who had haemodynamic instability from November 2012 to February 2020 that were seen at two academic teaching hospitals with roles as a burn centre and tertiary referral centre at the University of Texas Southwestern Medical Center in Dallas, TX USA. Patients with a discharge diagnosis of AGEP that had haemodynamic instability during their eruption were included. Patients with a history of psoriasis, presentations thought to be a flare of generalized pustular psoriasis, or concurrent infection during eruption were excluded. AGEP with haemodynamic instability was characterized by degree of hypotension, dermatologic phenotype at time of dermatologic consultation, and management approach.

**Results:**

This study included 19 patients with AGEP‐associated haemodynamic instability (mean age, 52 years; age range, 29–76 years; 11 (58%) female). Patients were classified on a spectrum of haemodynamic instability; three had sustained hypotension, 10 had hypotension with organ dysfunction, and six had shock. Patients with AGEP‐associated haemodynamic instability had a range of dermatologic phenotypes at initial consultation: subtle exanthematous eruption with minimal pustules, typical eruption with pustules and flexural predominance, and severe eruption with features of Stevens–Johnson syndrome. Both topical and systemic corticosteroids were used for treatment of several patients. Of the patients that required vasopressors and received systemic steroids, the majority were off vasopressors within 24 h of steroid initiation.

**Conclusion:**

Approximately 22% of patients presenting with AGEP to a tertiary referral center had haemodynamic instability. Clinicians should be aware that dermatologic phenotype of AGEP at presentation does not correlate with development of haemodynamic instability.

## Graphical abstract

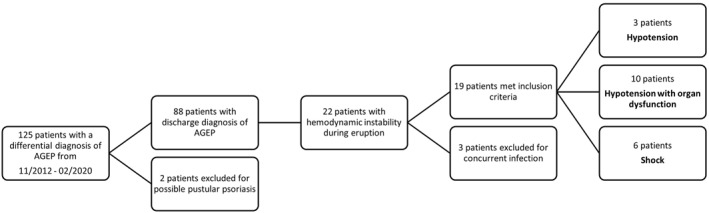

1

**What is already known about this topic?**

- Acute generalized exanthematous pustulosis (AGEP) is a severe cutaneous adverse reaction characterized by the acute onset of oedematous erythema in the intertriginous areas accompanied by pinpoint non‐follicular sterile pustules occurring hours to days after culprit drug initiation.
- AGEP with haemodynamic instability has rarely been described in the literature and two of the largest retrospective studies of AGEP with systemic involvement did not collect data related to blood pressure or hypotension.

**What does this study add?**

- In this retrospective case series, 22% of patients seen at our tertiary referral centres with AGEP had haemodynamic instability.
- Dermatologic phenotype at the time of dermatology consultation did not correlate with AGEP‐associated haemodynamic instability.
- Of the patients that required vasopressors and received systemic steroids, the majority were off vasopressors within 24 h of steroid initiation.

## 2 INTRODUCTION

Acute generalized exanthematous pustulosis (AGEP) is a severe cutaneous adverse reaction (SCAR). Approximately 90% of cases are precipitated by drugs, especially antibiotics including penicillins, macrolides, quinolones, and sulfonamides, although infectious etiologies have also been described.^1^, ^2^, ^3^

Acute generalized exanthematous pustulosis is characterized by the acute onset of oedematous erythema in the intertriginous areas accompanied by pinpoint non‐follicular sterile pustules occurring hours to days after culprit drug initiation.^4^ Other skin findings, such as facial oedema, atypical targetoid lesions, and vesicles have been described, but are not common for AGEP.^4^ AGEP is associated with fever and leucocytosis, however systemic involvement, including renal, hepatic, and pulmonary, is rare.^5^, ^6^, ^7^ While AGEP is rapidly progressive, there is a low risk of mortality and overall prognosis is favourable with quick resolution upon cessation of the causative agent.^4^, ^8^

Although systemic involvement of AGEP is uncommon, there are rare reports of AGEP‐associated haemodynamic instability in the literature.^9^, ^10^, ^11^, ^12^, ^13^, ^14^, ^15^, ^16^, ^17^, ^18^, ^19^, ^20^, ^21^, ^22^, ^23^ These reports document cases of AGEP with hypotension requiring intravenous fluid resuscitation and some necessitating vasopressor support.^9^, ^10^, ^11^, ^12^, ^13^, ^14^, ^15^, ^16^, ^17^, ^18^, ^19^, ^20^, ^21^, ^22^, ^23^ Since AGEP with haemodynamic instability is rarely encountered, there is a lack of information regarding dermatologic presentation, laboratory findings, and treatment of these patients. Furthermore, two of the largest retrospective studies documenting systemic involvement of AGEP did not collect data related to blood pressure or hypotension.^5^, ^6^ Here, we present a series of 19 cases of AGEP with haemodynamic instability and summarise the clinical and dermatologic characteristics, management, and outcomes in this uncommon presentation of AGEP.

## 3 MATERIALS AND METHODS

**FIGURE 1:**
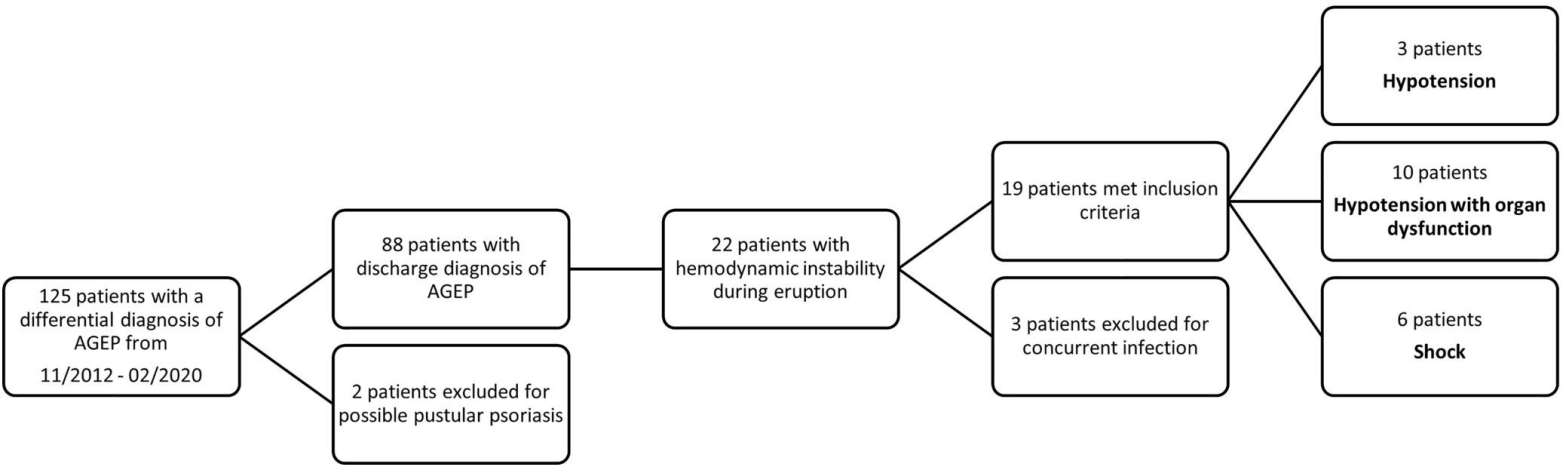
Flow diagram of patient identification and haemodynamic instability classification

A retrospective IRB approved study was performed using the dermatology inpatient registry for the two academic teaching hospitals at UT Southwestern Medical Center, and adult hospitalized patients diagnosed with AGEP between November 2012 and February 2020 were identified. Only patients with a discharge diagnosis of AGEP by the inpatient dermatology service, retrospectively validated by the European Study of Severe Cutaneous Adverse Reactions (EuroSCAR) scoring system with ≥5 points indicating probable or definite AGEP, were included in the study.^4^ Patients with a history of psoriasis or flares of generalized pustular psoriasis were excluded from the study, as well as those with other severe cutaneous adverse reactions, such as Stevens–Johnson syndrome (SJS)/toxic epidermal necrolysis, and drug reaction with eosinophilia and systemic symptoms. For patients that presented phenotypically like SJS, the diagnosis of AGEP was confirmed through histopathologic evaluation. Data were collected for patients with haemodynamic instability, defined as two sustained low blood pressure readings of systolic blood pressure <90 mmHg or drop >40 mmHg. Patients were further classified (Figure 1) with sepsis criteria on organ dysfunction and shock criteria for those not responsive to fluid resuscitation.^24^ Infectious and cardiogenic etiologies of shock were ruled out by negative cultures and transthoracic echocardiogram, the latter of which was reviewed when deemed to be clinically indicated.

Electronic medical records of patients seen at Parkland Health and Hospital System and University of Texas Southwestern Medical Center Affiliated Hospitals were reviewed for demographics, past medical history, clinical presentation, laboratory results, histopathologic evaluation, treatment course, and outcomes.

## 4 RESULTS

Among 88 hospitalized patients with a discharge diagnosis of AGEP between November 2012 and February 2020, 19 had AGEP‐associated haemodynamic instability. Table 1 summarises the demographics and cutaneous findings at time of dermatology consultation of these 19 patients. The age at diagnosis ranged from 29 to 76 years, with a mean of 52.0 ± 15.1 years.

**TABLE 1:** Characteristics of patients with AGEP‐associated haemodynamic instability

Characteristic	Finding (*N* = 19)^a^
Age at presentation, mean (years)	52.0
BMI (kg/m^2^), mean	34.4
Sex	
Female	11 (58)
Male	8 (42)
Race	
White, non‐Hispanic	7 (37)
Black, non‐Hispanic	7 (37)
Hispanic	5 (26)
Past medical history	
Hypertension	11 (58)
Multiple comorbidities	11 (58)
Diabetes	6 (32)
Uncontrolled	4 (21)
Heart disease	6 (32)
Heart failure with preserved ejection fraction	5 (26)
Arrhythmia	4 (21)
Congenital	1 (1)
Lung disease (COPD or asthma)	7 (37)
Suspected trigger	
Antibiotic	10 (53)
Calcium channel blocker	2 (11)
Multiple	2 (11)
Other^b^	4 (21)
Unknown	1 (5)
Presumed source of hypotension prior to AGEP diagnosis, (*n* = 15)	
Urinary tract infection	5 (33)
Abdominal infection	4 (27)
Pneumonia	4 (27)
Other^c^	2 (13)
Initial dermatologic findings	
Pustules	17 (89)
Exanthematous eruption	4 (21)
Skin erosions	3 (16)
Bullae	6 (32)
Positive Nikolsky's sign	3 (16)
Atypical targetoid lesions	3 (16)
Facial oedema	4 (21)
Erythroderma	3 (16)
Superficial desquamation	8 (42)

Abbreviations: AGEP, acute generalized exanthematous pustulosis; BMI, body mass index; COPD, chronic obstructive pulmonary disease.

^a^ Data are presented as number (percentage) of patients unless otherwise indicated.

^b^ Suspected triggers include NSAID, antifungal, antihistamine, antidepressant.

^c^ Other presumed sources of hypotension include cellulitis and endocarditis.

At initial dermatology evaluation, four (21%) patients had subtle skin findings, presenting with an exanthematous eruption and minimal pustules, eight (42%) patients had findings typical for AGEP, and seven (37%) patients had a severe presentation with features of SJS, including bullae, skin erosions, or positive Nikolsky's sign; they did not have mucosal involvement. Seventeen (89%) patients had pustules present at consultation, and two (11%) patients had only an exanthematous eruption but developed pustules the following day.

Prior to steroid initiation, neutrophilia was seen in 95% of patients. The mean white blood cell count (WBC) was 18.0 × 10^9^/L (range: 7.0–61.1 × 10^9^/L, normal: 4.5–11.0 × 10^9^/L), and the mean absolute neutrophil count (ANC) was 15.3 × 10^9^/L (range: 4.3–58.7 × 10^9^/L, normal: 1.8–7.7 × 10^9^/L). Ten (53%) patients had an elevated blood lactate level, with a mean lactate of 3.3 mmol/L (range: 2.1–5.0 mmol/L, normal: <2.0 mmol/L). Nine (47%) patients were febrile. With regards to systemic involvement of AGEP, ten (53%) patients had an acute kidney injury, four (21%) patients had pulmonary involvement, and one (5%) patient had hepatocellular involvement.

The management and outcomes of the cohort are outlined in Table 2. Six (32%) patients required vasopressors, while hypotension resolved with fluid resuscitation in thirteen (68%) patients. For the patients that received systemic steroids, the drug, dose, and duration is recorded in Table 2. Of the patients that required vasopressors and received systemic steroids and excluding the patient who was continued on vasopressors for reasons other than hypotension, three (75%) were weaned off vasopressors within 24 h of systemic steroid initiation. Fifteen (79%) patients were also managed with empiric antibiotics, and five (26%) patients received empiric antifungal medication.

**TABLE 2:** Management and outcomes of individual patients with AGEP‐associated haemodynamic instability

Haemodynamic instability classification	Skin phenotype at dermatology consultation^a^	Steroid treatment^b^	Time on systemic steroids to pressor cessation (h)	Initial systemic treatment	Dose (mg/d)	Duration (days)	Subsequent systemic treatment	Dose (mg/d)	Duration (days)	Time from dermatology consultation to discharge (days)
Hypotension	Subtle	Combined	–	Prednisone	40	14	–	–	–	5
	Severe	Topical	–	Cyclosporine	250	7	–	–	–	4
	Severe	Topical	–	–	–	–	–	–	–	6
Hypotension with organ dysfunction	Subtle	Combined	–	Methylprednisolone	240	2	Prednisone	80	11	7
	Typical	Topical	–	–	–	–	–	–	–	5
	Typical	Combined	–	Methylprednisolone	120	3	Prednisone	60	6	5
	Typical	Topical	–	–	–	–	–	–	–	9
	Typical	Topical	–	–	–	–	–	–	–	19
	Typical	Topical	–	–	–	–	–	–	–	6
	Severe	Combined	–	Methylprednisolone	160	3	–	–	–	6
	Severe	Combined	–	Methylprednisolone	240	2	Prednisone	60	16	10
	Severe	Topical	–	–	–	–	–	–	–	8
	Severe	Topical	–	–	–	–	–	–	–	9
Shock	Subtle	Combined	7.6	Hydrocortisone	200	1	Methylprednisolone	120	7	15
	Subtle	Topical	–	–	–	–	–	–	–	8
	Typical	Systemic	5.0	Hydrocortisone	200	1	Methylprednisolone	80	2	15
	Typical	Combined	3.7	Methylprednisolone	375	2	Prednisone	60	6	4
	Typical	Combined	208.3^c^	Methylprednisolone	500	5	Prednisone	60	9	13
	Severe	Combined	25.0	Methylprednisolone	160	6	–	–	–	14

Abbreviation: AGEP, acute generalized exanthematous pustulosis.

^a^ Subtle phenotype: exanthematous eruption with minimal pustules or early in disease progression; typical phenotype: eruption with flexural predominance and pustules; severe phenotype: eruption with features of Stevens‐Johnson syndrome.

^b^ Patients with combined steroid treatment received both systemic corticosteroids and topical triamcinolone ointment 0.1%.

^c^ Patient's prolonged pressor duration due to continued low dose dopamine for acute kidney injury and pulmonary hypertension.

Fourteen (74%) patients were admitted to the Intensive Care Unit (ICU) for AGEP‐associated haemodynamic instability, with a mean ICU duration of 5 days (range: 2–13 days). For all study patients, the mean duration of hospital stay from dermatology consultation to discharge was 10 days (range: 4–19 days).

## 5 DISCUSSION

Acute generalized exanthematous pustulosis is a SCAR characterized by fever, pinpoint pustules on a background of erythema, and neutrophilia.^4^ AGEP is generally self‐resolving with cessation of the causative agent and treatment is mainly supportive.^8^ Systemic involvement of AGEP has been described, most commonly involving the liver and kidneys and less often the lungs.^5^ However, only 15 adult cases of AGEP with haemodynamic instability have been reported in the literature.^9^, ^10^, ^11^, ^12^, ^13^, ^14^, ^15^, ^16^, ^17^, ^18^, ^19^, ^20^, ^21^, ^22^, ^23^

The pathophysiology of AGEP is a drug‐specific CD4+ and CD8+ T cell response that results in increased production of IL‐8, a potent neutrophil‐attracting chemokine.^25^ Other inflammatory cytokines, including IL‐17 and IL‐22, also have a role in the pathophysiology of AGEP, and may be involved in the systemic complications of AGEP.^25^, ^26^ These cytokines participate in neutrophil chemotaxis, tissue homeostasis, and coordination of the systemic inflammatory response through their influence on chemokine production.^26^, ^27^ Through triggering further production of other pro‐inflammatory mediators, both IL‐17 and IL‐22 may have a role in inducing cytokine storm with resultant haemodynamic instability and multi‐organ dysfunction. Additionally, the downstream effects of excess cytokine release can result in endothelial barrier dysfunction, contributing to a possible capillary leak syndrome.^24^ While the pathophysiology of AGEP‐induced haemodynamic instability is likely multifactorial, these cytokines may contribute to the pathologic findings of systemic involvement, including capillary leak syndrome, distributive shock and subsequent organ injury.

Approximately 22% of hospitalized patients diagnosed with AGEP from November 2012 to February 2020 had haemodynamic instability. Of those with haemodynamic instability, 36% met shock criteria. A previous study of 28 AGEP cases reported haemodynamic instability in 7% of their patients.^7^ This difference may be due to our two hospitals with roles as a burn centre and tertiary referral centre, as well as management in the inpatient setting.

Regarding underlying medical conditions of patients with AGEP‐induced haemodynamic instability, obesity, hypertension, and the presence of multiple comorbid conditions was common. Other retrospective studies on AGEP have reported similar findings, with one study noting an increased BMI in patients diagnosed with severe cases of AGEP.^7^, ^28^ The dermatologic findings for these patients ranged from an exanthematous rash with subtle pustules to a severe phenotype with features of SJS. These results indicate that the presentation of AGEP at initial dermatology evaluation can vary in the acute setting.^4^ The severity of dermatologic phenotype at initial consultation did not determine the development of AGEP‐related haemodynamic instability. It is imperative that clinicians closely monitor patients who present with morbilliform exanthem and hypotension and have a low threshold to biopsy patients looking for AGEP, as the morphology of these lesions can change rapidly.

All but four patients had a presumed diagnosis of sepsis and were initially managed with empiric antibiotics. At dermatology consultation, the clinical picture was consistent with AGEP, and the suspected causative agent had already been discontinued. Since AGEP is thought to have a benign and self‐limited course after removal of the suspected trigger, systemic corticosteroid treatment is typically not indicated.^4^ However, systemic steroids were warranted in many cases across the haemodynamic instability spectrum, with a predominance in the shock‐like AGEP group. Notably, the patients that required vasopressors for management of their AGEP‐related shock and received systemic steroids had a marked clinical response to steroids, with the majority weaned off vasopressors within 24 h of steroid initiation. Furthermore, a patient that was on vasopressors for 4 days prior to receiving systemic treatment responded quickly to systemic steroids with cessation of vasopressors 5 h after steroid initiation. Of the patients receiving systemic corticosteroids, one developed steroid‐induced hyperglycemia that resolved within two days; no other side effects were noted in other patients. Therefore, patients with AGEP‐induced shock may benefit from early initiation of systemic steroids. With regards to other systemic involvement of AGEP, acute kidney injury was commonly seen, and pulmonary involvement was also noted in a few patients. These findings correlate with other retrospective studies on AGEP.^5^, ^7^

## 6 CONCLUSIONS

AGEP with haemodynamic instability is rarely encountered.^5^, ^6^ Our study demonstrates that this presentation may be more common than what has been described previously as 22% of the patients at our tertiary referral centres have this complication. Additionally, severity of dermatologic phenotype at the time of dermatology consultation does not correlate with AGEP‐associated haemodynamic instability. As such, AGEP should be in the differential diagnosis in patients with exanthematous eruptions and hypotension, as early pustules may be subtle. Given the marked clinical improvement and cessation of vasopressors within 24 h of systemic steroid initiation in our patients with shock, patients with AGEP‐induced shock may benefit from treatment with systemic steroids.

Limitations of this study are its retrospective nature, inclusion of inpatient data from only two hospitals, and the small sample size. Further studies including patients treated successfully in the outpatient setting are needed to examine if certain dermatologic phenotypes of AGEP are more prone to systemic involvement and haemodynamic instability. Additionally, research is needed on the use of systemic steroids in the treatment of severe presentations of AGEP, and future studies examining AGEP‐associated shock may further elucidate the benefit of early initiation of systemic steroids and time to cessation of vasopressors.

## CONFLICTS OF INTEREST

None reported for all authors.

## AUTHOR CONTRIBUTIONS

**M. O’Brian:** Conceptualization; Investigation; Methodology; Writing – original draft; Data curation; Formal analysis; Writing – review & editing. **C. Thomas:** Conceptualization; Methodology; Writing – review & editing. **A. R. Dominguez:** Conceptualization; Methodology; Writing – review & editing. **M. Mauskar:** Conceptualization; Formal analysis; Methodology; Writing – review & editing.

## Data Availability

Data available on request from the authors.
